# Quality of Life and Psychological Disorders in Coeliac Disease: A Prospective Multicentre Study

**DOI:** 10.3390/nu13093233

**Published:** 2021-09-16

**Authors:** Cristina Canova, Isabella Rosato, Ilaria Marsilio, Flavio Valiante, Valerio Zorzetto, Giovanni Cataudella, Anna D’Odorico, Fabiana Zingone

**Affiliations:** 1 Unit of Biostatistics, Epidemiology and Public Health, Department of Cardio-Thoraco-Vascular Sciences and Public Health, University of Padua, 35131 Padova, Italy; cristina.canova@unipd.it (C.C.); isabella.rosato@studenti.unipd.it (I.R.); 2 Department of Surgery, Oncology, Gastroenterology, University of Padua, 35124 Padova, Italy; ilaria.marsilio@gmail.com (I.M.); anna.dodorico@aopd.veneto.it (A.D.); 3 Gastroenterology and Digestive Endoscopy Unit, ULSS 1 Dolomiti, Santa Maria del Prato Hospital, 32032 Feltre, Italy; flavio.valiante@aulss1.veneto.it; 4 Gastroenterology and Endoscopy Unit, ULSS 9, 37045 Legnago, Italy; valerio.zorzetto@aulss9.veneto.it; 5 Gastroenterology and Endoscopy Unit, San Bortolo Hospital, 36100 Vicenza, Italy; giovanni.cataudella@aulss8.veneto.it

**Keywords:** celiac disease, quality of life, depression, anxiety, dietary compliance

## Abstract

Coeliac disease (CeD) has been associated with psychological disorders and reduced quality of life. Our prospective study evaluated the changes in the quality of life, anxiety and depression in CeD patients up to two years after diagnosis. We recruited adult patients residing in the Veneto region with a new diagnosis of CeD. Several validated questionnaires were administered to measure quality of life, psychological symptoms and adherence to a gluten-free diet (GFD) at the time of diagnosis and after 1 and 2 years. Ninety-three patients reached the 1-year follow-up (81.7% were females with a median age at diagnosis of 35 years), and 55 patients reached the 2-year follow-up. We observed a significant improvement in quality of life, anxiety and depression scores at 1 year after diagnosis, particularly in patients who complied with a GFD. The improvements among classical CeD patients were similar to those observed in nonclassical patients except for anxiety, which improved only in patients with a classical presentation at diagnosis. Age, sex and other disease factors did not affect the change in quality of life (QoL) or other mood disorders. Most of the improvements measured 1 year after diagnosis and 2 years after diagnosis were not significant. In conclusion, QoL and mood disorders must be considered, and psychological counselling should be used when needed.

## 1. Introduction

Coeliac disease (CeD) is a chronic immune-mediated disease characterised by autoantibody production and intestinal villous atrophy generated by gluten ingestion in genetically predisposed individuals [1]. Several reports have focused their attention on the difficulties of living with CeD, particularly regarding its impact on physical, social and emotional factors in adults [2]. Quality of life (QoL) generally improves on a gluten-free diet (GFD) [2]. This is particularly true in symptomatic patients at diagnosis [3,4,5,6], even if some studies described a similar improvement in screening-detected patient [4]. Nachman et al. described the most significant QoL improvement in the first three months after starting a GFD, with some additional improvement up to one year later [3]. The continuation of this latter study described a significant QoL deterioration at 4 years postdiagnosis compared with 1 year, even if the scores remained significantly better than those at diagnosis, and observed a long-term impairment of the QoL in patients who did not strictly comply with a GFD [7]. One year later, Barratt et al. underlined that the main contributing factor to a reduced QoL was not dietary compliance but the perceived degree of difficulty of adhering to a GFD, influenced by social and educational background [8]. A recent systematic review with a meta-analysis [9] concluded that a GFD significantly improves but does not normalise the QoL in adults with CeD, particularly in symptomatic patients, and that dietary adherence improves QoL. A cross-sectional Italian study described an overall good QoL in 100 CeD patients on a GFD with patients with good compliance with a GFD tending to have a better QoL while patients who did not comply with a GFD appeared to suffer from dysphoria (*generalised dissatisfaction with life*) [10]. However, it is always difficult to understand the cause and the effect among low compliance with a GFD and low QoL. Several psychosocial factors are described in CeD adult patients [2], and these factors may influence the QoL of CeD patients, both reducing psychological well-being and indirectly reducing adherence to a GFD [11]. In addition, other determinant factors can affect both QoL and mood disorders such as anxiety and depression in CeD patients, demographic factors such as age, gender, education level, employment status, and disease-related factors such as the presence of comorbidities and disease duration [12,13,14].

However, the current literature on these topics has several limitations in terms of a small sample size and study design; therefore, this prospective observational multicentre study was designed to evaluate the changes in QoL, anxiety and depression in CeD adult patients up to two years after diagnosis. We also investigated the effect of sociodemographic, clinical and behavioural factors on these changes.

## 2. Methods

### 2.1. Population and Study Design

This multicentre longitudinal study was conducted in collaboration with the Italian Association of Coeliac Disease Veneto. All adults (age >18 years old) with a new CeD diagnosis who resided in the Veneto region were consecutively enrolled by a gastroenterologist in each of the four specialised centres for diagnosis and CeD treatment in the region involved in the study. Details of the study protocol and the sociodemographic, behavioural and clinical profiles at diagnosis have been described elsewhere [15].

The diagnosis of CeD was based on duodenal atrophy using the Marsh classification associated with positive anti-tissue transglutaminase/endomysial IgA antibodies. In a case of IgA deficiency or in a case of seronegative CeD, details on IgG serology and HLA testing must be specified.

After written consent, patients were assessed both at the time of diagnosis and during 4 planned follow-up medical examinations at 12, 24, 36, and 48 months using structured and validated questionnaires. In this paper, we focused on the recruited patients with at least information on the 12-month follow-up (T1). A subset of patients with a 24-month follow-up (T2) were analysed.

### 2.2. Procedures and Clinical Characteristics of Patients

At the time of diagnosis, we collected data on sociodemographic characteristics, diagnostic path, clinical characteristics including types, the duration of symptoms before diagnosis, and the presence of other pathologies (associated or not with coeliac disease). Moreover, copies of the biopsy reports, antibody assays and routine blood test results were collected.

According to the clinical status of the patients at the time of diagnosis, they were categorised as presenting either classical symptomatic disease (signs and symptoms of malabsorption, especially diarrhoea and weight loss) or nonclassical symptomatic disease (without any signs and symptoms of malabsorption but with at least one other symptom/sign of CeD). We separately considered patients with anaemia without any other classical symptoms/signs; and finally, we defined asymptomatic CeD patients as those who did not report any symptoms/signs.

Gastrointestinal (GI) and extraintestinal (EI) symptoms were collected at each follow-up using the same questionnaire by the gastroenterologist. At the end of the visit, the same physician provided a package of self-completed questionnaires that had to be returned soon afterwards to all patients.

### 2.3. Assessment of the Compliance with a Gluten-Free Diet

Adherence to a GFD was assessed at each follow-up with a validated score developed by Biagi et al. [16]. The score is based on just four simple questions that can be administered in less than a minute, even by nonexpert personnel.

The Biagi scores (ranging from 0 to 4) were categorised. Patients not following a GFD (with a score equal to 0–1) or following a GFD but with important errors (with a score equal to 2) were grouped as nonadherent patients, and patients following a strict GFD (with a score equal to 3–4) were grouped as adherent patients.

### 2.4. Self-Administered Quality of Life and Psychological Symptoms Questionnaires

QoL and PS (psychological symptoms) were assessed both at diagnosis and at different times using a set of self-administered scales, as previously reported [15].

The Beck Depression Inventory (BDI) is used as a psychometric assessment of depression [17,18,19]. The instrument involves 21 items with four response alternatives (0–3).

The State-Trait Anxiety Inventory (STAI) is a commonly used measure of trait and state anxiety. It can be used in clinical settings to diagnose anxiety and to distinguish it from depressive syndromes. Its most popular version has 20 items for assessing trait anxiety and 20 items for assessing state anxiety. All items are rated on a 4-point scale (e.g., from “almost never” to “almost always”) [20,21,22].

The HADS (Hospital Anxiety Depression Scale) questionnaire is divided into two parts to investigate the presence of anxiety (HADS-A) and depression (HADS-D) symptoms in patients with organic pathologies. The questionnaire involves a total of 14 items, and each item is rated on a 3-point scale [23,24].

The Short Form Health Survey (SF-36) is a 36-item, patient-reported survey of patient health. The SF-36 consists of eight scaled scores, which are the weighted sums of the questions in each domain. Each scale is directly transformed into a 0–100 scale on the assumption that each question carries equal weight. The lower the score is, the worse the disability. A score of zero is equivalent to the maximum disability, and a score of 100 is equivalent to no disability [25].

All the questionnaires mentioned above were presented to patients using Italian language validated versions.

### 2.5. Statistical Analysis

Descriptive statistics were used to describe sociodemographic and clinical characteristics and QoL scores. The descriptive statistics reported were the mean, median, and interquartile range for continuous variables and frequencies and percentages for categorical variables. Most variables in our analysis did not follow a normal distribution, as assessed by the Shapiro–Wilk test. Therefore, we chose all nonparametric statistics.

To assess differences in QoL and psychological symptoms at diagnosis and after 12 months, the Wilcoxon signed rank test was used.

Assessments of the score changes at the first-year follow-up in patients according to their gender, age classes (18–34 and 35 or above), CeD presentation at diagnosis (classical and nonclassical), and adherence to a GFD (at T1) were performed using Mann–Whitney nonparametric tests.

Differences between scores at the three follow-up times (T0, T1, and T2) were estimated using Friedman’s nonparametric test and Dunn post hoc test, adjusted by Bonferroni for multiple comparisons.

Univariate and multivariate analyses were performed, after the scores reported on the questionnaires and the number of symptoms were dichotomised as “improved score” or “not improved score” categories. The categorisation was based on the difference in the scores between T1 and T0 as well as the difference in the number of GI and EI symptoms between T1 and T0. Patients with no variation in the score results and in the number of symptoms were included in the category of “not improved score”. Adherence to GFD, type of CeD, gender and age were included in a logistic regression model as potential predictors for not improvement in the questionnaires’ scores and the number of GI and EI symptoms. The results are shown as unadjusted odds ratios (ORs) and adjusted odds ratios (aORs) with 95% confidence interval (CI). A *p*-value < 0.05 was considered significant. All analyses were performed using R 4.0.2 (R Development Core Team 2010, R Foundation for Statistical Computing, Vienna, Austria. ISBN 3-900051-07-0, URL: http://www.R-project.org/, accessed on 10 September 2021).

### 2.6. Ethical Considerations and Good Clinical Practice

The study protocol was approved by the Research and Ethical Committees of each hospital involved (prot. n. 10623, 24 February 2016). Each patient was included in the study only after providing his or her written informed consent, and participants were allowed to withdraw from the study for any reason at any time. Personal data were processed according to Legislative Decree 196/2003. The study was conducted in compliance with current laws and decrees.

## 3. Results

From April 2016 to January 2020, a series of 114 adults with a new diagnosis of CeD were enrolled, and 110 individuals (96.49%) agreed to participate in the study and signed the consent form (98 women and 21 men). Ninety-three of these patients (85%) completed the first follow-up (April 2017–November 2020), and 55 patients (48.2%) completed the second follow-up (April 2018–July 2021).

### 3.1. Sociodemographic and Clinical Characteristics at Diagnosis

The sociodemographic and clinical characteristics of the recruited subjects who completed the first (*n* = 93) and second follow-ups (*n* = 55) are presented in Table 1. All the presented information was retrieved at the baseline. Most of the participants included in the main analyses (T0–T1) were women (*n* = 76, 82%), and the average age was 37.3 years (SD 13.2). Similarly, most of the patients included in the T0, T1, and T2 analyses were women (*n* = 47, 85%) with an average age of 39.6 years at diagnosis (SD 13.7).

At diagnosis, 46 patients presented classical CeD (*n* = 46), and 47 patients presented nonclassical CeD (*n* = 47). We decided to consider asymptomatic patients with nonclassical CeD due to the small number of subjects in this subgroup (*n* = 2). In completely asymptomatic patients, the diagnosis of CeD was achieved after performing a screening test; both patients reported the presence of other cases of CeD in the family.

Positivity for IgA tissue transglutaminase antibodies was detected in 96.7% of patients with three patients identified as seronegative (negative CeD serology, duodenal atrophy and HLA-DQ2 positivity). Villous atrophy was found in 91.4% of patients while 8.6% had minor lesions consistent with potential coeliac disease. All patients with potential CeD had both anti-transglutaminase IgA and anti-endomysium IgA positivity, were HLA DQ2- or DQ8-positive, and were symptomatic. No patient had IgA deficiency.

### 3.2. Clinical and Biochemical Response and Compliance with a GFD

As presented in Supplementary Materials Table S1, we observed a significant decrease in the number of gastrointestinal symptoms among the large majority of patients after the 1-year follow-up (68/93, 73%). Similar results were obtained when considering extraintestinal symptoms with 75 patients (81%) showing a reduction in the number of extraintestinal symptoms at T1. Considering the subgroup of patients who provided information at T2, we observed (according to the previous observations) a reduction of symptoms from T0 to T1 (in 42 patients for gastrointestinal symptoms and in 44 patients for extraintestinal symptoms) and from T0 to T2 (in 37 patients for gastrointestinal symptoms and in 45 patients for extraintestinal symptoms) (Supplementary Materials, Table S1).

Information about IgA transglutaminase antibody levels was collected for a total of 70 patients at both T0 and T1. At T0, 69 of them showed positivity for IgA antibodies while 23 out of 69 (33.3%) were still positive at T1, mostly presenting a value very near the normal threshold. Considering the subgroup that provided complete information about IgA antibody levels at T0 and T2, we observed that at T0, all 46 patients showed positivity for IgA antibodies while 8 patients out of 46 (17.4%) were still positive at T2.

At T1, based on Biagi’s questionnaire, most of the patients reported strict adherence to a GFD (*n* = 78, 87%). Of these patients, 2.2% followed a GFD with relevant errors that require correction, and 11.1% did not adhere to a GFD at all. Similar percentages were seen at T2 with 48 (90.6%) patients following a GFD correctly, 1.9% following a diet with important errors and 7.5% not adhering to a GFD at all. No significant difference in diet adherence was observed according to CeD presentation at diagnosis, although GFD adherence was higher (88.9%) in classical than nonclassical (84.4%) individuals (*p* = 0.756 using the χ^2^ test with continuity correction).

No patient underwent an upper endoscopy during the follow-up.

### 3.3. Psychological Aspects and QoL

At T1, all the CeD patients’ QoL and psychological symptom scores significantly improved from the baseline (Table 2).

**Figure 1:**
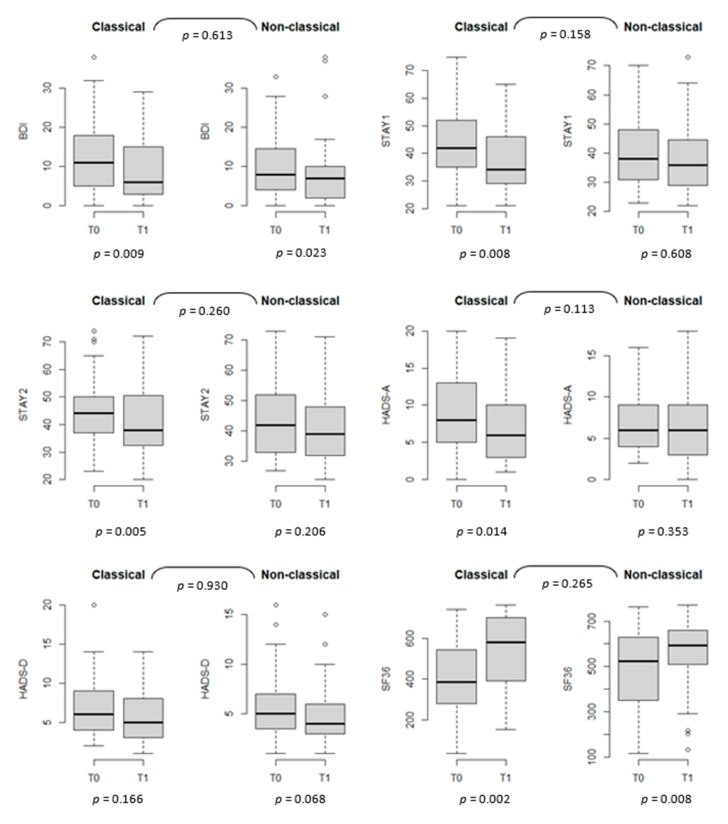
Quality of life and psychological scales at T0 and T1, stratified by type of celiac disease (CeD), *n* = 93. BDI: Beck Depression Inventory; STAI 1, STAI 2: State-Trait Anxiety Inventories; HADS-A, HADS-D: Hospital Anxiety and Depression Scales; SF36: Short Form Health Survey.

Group comparisons of the QoL and psychological symptoms over time between classical and nonclassical CeD patients are shown in Figure 1 (Supplementary Materials, Tables S2–S4). The improvements among classical CeD patients were not significantly better than the improvements among nonclassical CeD patients except for anxiety scores (STAI 1 and HADS-A), which improved only in the patients with classical presentation at diagnosis (with *p* = 0.008 and *p* = 0.014, respectively).

**Figure 2:**
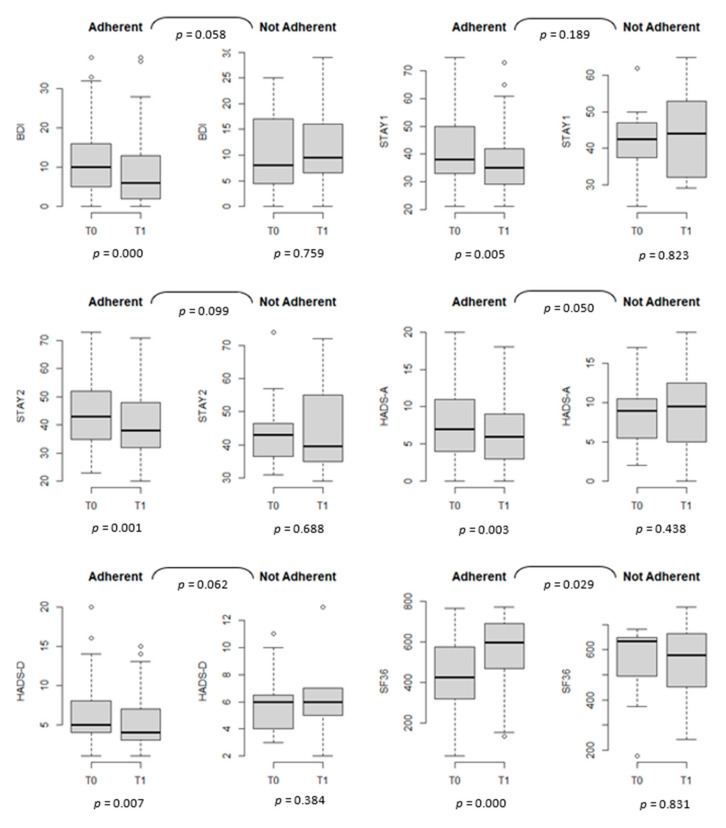
Quality of life and psychological scales at T0 and T1, stratified by adherence to a gluten-free diet (GFD), *n* = 93.

The QoL and psychological symptoms over time between adherents (*n* = 78) and nonadherents to a GFD (*n* = 12) are shown in Figure 2 (Supplementary Materials, Tables S2 and S4). The improvements in anxiety and depression scores among the adherents to a GFD were significantly (or reached statistical significance) better than those among nonadherents. QoL, measured through the SF-36 scale, worsened in nonadherent subjects (with *p* = 0.000 for adherent patients and *p* = 0.831 in nonadherent patients).

There were no sex or age differences in psychological/depression scores or QoL over time (Supplementary Materials, Tables S3 and S4).

In the analyses restricted to the 55 subjects with a 24-month follow-up, all the QoL and anxiety and depression scores, except STAI 1 (*p* = 0.063), were significantly different at the different time points (Table 3). All scores improved during the interval between T0 and T1 with either maintenance or discrete improvement between T1 and T2 (Supplementary Materials, Table S5).

Multivariate analysis showed adherence to GFD to be the most relevant factor influencing the worsening of patients’ conditions, in accordance with the results already reported (adjusted OR 4.05, CI 95% 1.09–14.99 for BDI, adjusted OR 4.14, CI 95% 1.03–16.75 for HADS-A); moreover, gender, age and type of CeD were not significantly associated with the absence of improvement in the patients’ conditions (Supplementary Materials, Table S4). It is relevant to underline that reported adjusted ORs do not differ much from the unadjusted ORs (Table 4).

## 4. Discussion

CeD is a chronic disease; and since lifelong adherence to a GFD is demanding and costly, the psychological aspects of these patients are affected. In our study, we found that QoL and mood disorders improved after CeD diagnosis, particularly in patients who complied with a GFD. The improvements among classical CeD patients were similar to those observed in nonclassical patients, except for anxiety, which improves only in patients with a classical presentation at diagnosis. This appears to be related to the fact that non-classical symptoms are as debilitating as classical symptoms. Age, sex and other disease factors did not seem to affect the change in QoL or other mood disorders.

Our results are mostly in line with previous literature regarding the positive effects of a GFD on QoL, anxiety and depression [7,10,26]. There is evidence of the persistence of depressive symptoms, possibly due to dietary restrictions that impair patients’ social relationships [27,28,29]. Therefore, noncompliance with a GFD might be either a consequence or a cause of persistent depression in CeD patients [30]. Hauser et al. described a higher level of anxiety and not depression in females on a GFD compared to the general population [31], and Addolorato et al. [29] reported that anxiety and not depression improves after one year on a GFD; however, no differences based on clinical presentation were described in these studies.

Our results did not describe a difference in QoL and depression improvement between those who had a classical or nonclassical presentation of the disease. Regarding this aspect, there are contradictory results available. Most of the studies compared symptomatic and screening-detected patients [2] without specifying those with classical and nonclassical symptoms. A recent systematic review and meta-analysis [9] reported that adherence to a GFD significantly improves but does not normalise the QoL in patients who were symptomatic at diagnosis [9].

Regarding the number of gastrointestinal and extraintestinal symptoms, we did not observe a significantly better improvement in adherent patients than in nonadherent patients. Most of our patients reported negative anti-transglutaminase IgA antibodies at the first and second follow-ups. No patient underwent a follow-up upper endoscopy, as suggested by the recent guidelines, since none had any suspected complications [32]. Most of the improvements measured from T1 to T2 were not significant (Supplementary Materials, Table S5), meaning that the positive effects observed in the patients were considerably related to the first period after diagnosis and then flattened over time, in accordance with previous studies [3] that showed that larger improvements occurred during the first three months of GFD adherence after diagnosis. Little is known about the long-term psychological outcomes of CeD patients over a large period of time, while our results were derived from a prospective multicentre study including approximately one hundred patients, half of whom reached the two-year follow-up, and considered several patient- and disease-related aspects. No recall and selection biases were present.

However, some limitations must be considered. First, the sample size was as follows: ninety-three patients reached the first-year follow-up, and fifty-five patients reached the second-year follow-up. This is mainly because some patients did not undergo the follow-up visits due to the COVID-19 pandemic. Moreover, the pandemic may have influenced some answers related to psychological aspects. However, a recent paper described that CeD patients did not feel more vulnerable because they had CeD, and they did not worry much about the possible shortages of gluten-free food during the pandemic [33]. Finally, we only included a small number of asymptomatic patients (*n* = 2), so we cannot describe the effect of a GFD in this particular subclass of patients.

In summary, we had previously shown that at diagnosis, patients with a classical presentation had a lower QoL than nonclassical patients who were found to be more depressed; a longer duration of GI symptoms decreased the self-reported SF36 scores in the physical health, social functioning and general health domains; and women had an overall lower self-perceived QoL [15]. These aspects can be related to the fact that patients may express concerns about unexplained symptoms and may feel frustrated about repeated consultations that offer no adequate explanation of their problems before diagnosis; and then at the time of diagnosis, there may be concerns about a diagnosis of a long-term condition.

While on a GFD, we found an overall improvement of the QoL, but not in all patients and in particular not for anxiety in those patients who had nonclassical symptoms or were asymptomatic at diagnosis. Some psychological problems may lessen with time as knowledge of the condition improves and biochemical abnormalities are corrected; however, some patients may have ongoing concerns about coping with the diet and do not adhere to it, particularly when going out and in social interactions.

In conclusion, QoL and mood disorders must be considered, and psychological counselling should be used when needed. Particular attention has been given to dietary compliance. This study represents a starting point for future observations of CeD patients with the objective of closing the gap of knowledge that still characterises the association between QoL and this complex pathology.

## Figures and Tables

**Table 1:** Sociodemographic and clinical characteristics at diagnosis of patients included at 12 months (T1) and 24 months (T2) follow-up.

Sociodemographic Characteristics	N° Patients Included at T1 (*n* = 93)	N° Patients Included at T2 (*n* = 55)
	*n* (%)	*n* (%)
Sex		
Females	76 (81.72)	47 (85.45)
Males	17 (18.28)	8 (14.55)
Civil Status		
Not married	50 (54.35)	26 (48.15)
Married	39 (42.39)	27 (50.0)
Divorced	2 (2.17)	0 (0)
Widowed	1 (1.09)	1 (1.85)
Education		
Degree/PhD	27 (29.35)	17 (31.5)
Upper secondary education	51 (55.43)	30 (55.6)
Lower secondary education	13 (14.13)	7 (12.9)
Primary education	1 (1.09)	0 (0)
Type of employment		
Employee	57 (61.96)	35 (64.8)
Self-employed	8 (8.7)	5 (9.3)
Housewife	9 (9.78)	6 (11.1)
Unemployed	4 (4.35)	2 (3.7)
Retired	2 (2.17)	2 (3.7)
Student	12 (13.04)	4 (7.4)
Smoking habit		
No	75 (80.65)	48 (87.3)
Yes	10 (10.75)	4 (7.2)
Ex smoker	8 (8.6)	3 (5.5)
Who suggested the diagnostic path to patients?		
Patient/Family/Friends	13 (13.98)	5 (9.1)
General practitioner	35 (37.63)	26 (47.3)
Gastroenterologist	13 (13.98)	7 (12.7)
Other specialist physician	32 (34.41)	17 (30.9)
With family affected by CeD		
No	74 (79.57)	46 (83.6)
Yes	19 (20.43)	9 (16.4)
Cohabitation with celiac patients		
No	85 (92.39)	52 (96.3)
Yes	7 (7.61)	2 (3.7)
Age, years		
mean (SD)	37.33 (13.21)	39.64 (13.73)
min-max	16.36–78.32	19.44–78.32
Median (Q1–Q3)	34.88 (27.26–45.77)	42.77 (27.53–51.13)
BMI		
mean (SD)	22.31 (3.49)	22.79 (3.53)
min-max	16.94–36.06	18.42–36.06
Median (Q1–Q3)	21.54 (20.01–24.1)	22.06 (20.26–24.14)
Type of CeD *		
Classical	46 (49.46)	26 (47.3)
Non classical	47 (50.54)	29 (52.7)
Non classical with anemia	19 (40.4)	9 (31.0)

Note: Numbers can vary based on the presence of missing values. All the presented information were retrieved at baseline. * CeD: celiac disease.

**Table 2:** Quality of life and psychological scales at T0 and T1, *n* = 93.

	T0			T1			*p*-Value *
	Min–Max	Median	1st–3rd Qu.	Min–Max	Median	1st–3rd Qu.	
BDI	0.0–38.0	9.0	5.0–16.5	0.0–38.0	6.0	2.0–13.5	0.001
STAI 1	21.0–75.0	39.0	32.8–49.3	21.0–73.0	36.0	29.0–44.8	0.023
STAI 2	23.0–74.0	43.0	34.8–51.3	20.0–72.0	39.0	32.0–49.0	0.004
HADS-A	0.0–20.0	7.0	4.0–11.0	0.0–19.0	6.0	3.0–10.0	0.019
HADS-D	1.0–20.0	6.0	4.0–8.0	1.0–15.0	4.0	3.0–7.0	0.024
SF36	35.5–764.0	468.1	326.0–587.1	133.0–772.0	592.5	470.0–687.5	0.000

Note: BDI: Beck Depression Inventory; STAI 1, STAI 2: State-Trait Anxiety Inventories; HADS-A, HADS-D: Hospital Anxiety and Depression Scales; SF36: Short Form Health Survey. * Wilcoxon test.

**Table 3:** Quality of life and psychological scales at T0, T1 and T2, *n* = 55.

	T0			T1			T2			*p*-Value *
	Min–Max	Median	1st–3rd Qu.	Min–Max	Median	1st–3rd Qu.	Min–Max	Median	1st–3rd Qu.	
BDI	0.0–38.0	8.0	5.0–16.5	0.0–38.0	6.0	2.0–11.0	0.0–28.0	4.0	1.0–8.0	0.001
STAI 1	21.0–72.0	38.0	31.0–50.5	21.0–65.0	34.0	28.0–41.8	20.0–60.0	32.0	28.0–39.0	0.063
STAI 2	23.0–73.0	43.0	34.0–51.5	22.0–65.0	38.0	31.8–45.8	20.0–67.0	36.5	30.3–44.0	0.000
HADS-A	0.0–20.0	7.0	4.0–11.0	0.0–18.0	4.5	3.0–8.8	0.0–15.0	4.0	3.0–7.0	0.000
HADS-D	2.0–20.0	5.0	4.0–8.0	1.0–15.0	4.0	3.0–7.0	1.0–14.0	4.0	3.0–6.8	0.001
SF36	35.5–764.0	418.8	294.8–560.5	133.0–772.0	608.2	472.8–696.5	227.8–791.0	612.8	480.7–704.2	0.000

* Friedman test.

**Table 4:** Logistic regression analysis of factors predicting the absence of improvement in scores and number of reported symptoms from baseline to 1-yr follow-up.

		**BDI**			**STAI 1**			**STAI 2**		
Predictor		*n* (%) *	OR (CI 95%)	aOR (CI 95%)	*n* (%) *	OR (CI 95%)	aOR (CI 95%)	*n* (%) *	OR (CI 95%)	aOR (CI 95%)
adherence to GFD	non adherent	8/12 (67)	3.57 (0.99; 12.93)	4.05 (1.09; 14.99)	7/12 (58)	1.72 (0.50; 5.89)	1.68 (0.48; 5.83)	8/12 (67)	3.57 (0.99; 12.93)	3.89 (1.04; 14.47)
	Adherent	28/78 (36)			35/78 (45)			28/78 (36)		
type of CeD	non classical CeD	20/47 (43)	1.05 (0.46; 2.40)	0.94 (0.39; 2.26)	25/47 (53)	1.48 (0.65; 3.35)	1.41 (0.61; 3.28)	21/47 (45)	1.26 (0.55; 2.87)	1.08 (0.45; 2.60)
	classical CeD	19/46 (41)			20/46 (43)			18/46 (39)		
Age	35+	24/54 (44)	1.28 (0.55; 2.96)	1.18 (0.48; 2.87)	27/54 (50)	1.17 (0.51; 2.66)	1.08 (0.46; 2.53)	21/54 (39)	0.74 (0.32; 1.71)	0.64 (0.26; 1.54)
	16–34	15/39 (38)			18/39 (46)			18/39 (46)		
Gender	M	9/17 (53)	1.73 (0.60; 4.97)	2.28 (0.76; 6.83)	8/17 (47)	0.94 (0.33; 2.69)	1.07 (0.36; 3.13)	8/17 (47)	1.29 (0.45; 3.71)	1.57 (0.52; 4.71)
	F	30/76 (39)			37/76 (49)			31/76 (41)		
		**HADS-A**			**HADS-D**			**SF36**		
**Predictor**		*n* (%) *	OR (CI 95%)	aOR (CI 95%)	*n* (%) *	OR (CI 95%)	aOR (CI 95%)	*n* (%) *	OR (CI 95%)	aOR (CI 95%)
adherence to GFD	non adherent	9/12 (75)	4.31 (1.08; 17.18)	4.14 (1.03; 16.75)	9/12 (75)	3.69 (0.93; 14.66)	3.80 (0.94; 15.35)	6/12 (50)	2.54 (0.47; 8.75)	2.87 (0.81; 10.17)
	Adherent	32/78 (41)			35/78 (45)			22/78 (28)		
type of CeD	non classical CeD	25/47 (53)	1.61 (0.71; 3.67)	1.58 (0.66; 3.67)	22/47 (47)	0.81 (0.36; 1.82)	0.80 (0.34; 1.87)	15/47 (32)	1.07 (0.44; 2.58)	0.90 (0.36; 2.26)
	classical CeD	19/46 (41)			24/46 (52)			14/46 (30)		
Age	35+	25/54 (53)	1.55 (0.67; 3.56)	1.46 (0.60; 3.52)	28/54 (52)	1.26 (0.55; 2.87)	1.19 (0.50; 2.81)	15/54 (28%)	0.69 (0.28; 1.66)	0.68 (0.27; 1.73)
	16–34	19/39 (41)			18/39 (46)			14/39 (36%)		
Gender	M	7/17 (41)	0.74 (0.25; 2.14)	0.92 (0.30; 2.8)	8/17 (47)	0.89 (0.31; 2.55)	1.05 (0.36; 3.09)	7/17 (41)	1.72 (0.58; 5.09)	1.88 (0.61; 5.79)
	F	37/76 (49)			38/76 (50)			22/76 (29)		
		**GI symptoms**			**EI symptoms**					
Predictor		*n* (%) *	OR (CI 95%)	aOR (CI 95%)	*n* (%) *	OR (CI 95%)	aOR (CI 95%)			
adherence to GFD	non adherent	4/12 (33)	1.36 (0.37; 4.98)	1.26 (0.32; 4.89)	4/12 (33)	2.29 (0.60; 8.66)	2.31 (0.57; 9.46)			
	Adherent	21/78 (27)			14/78 (18)					
type of CeD	non classical CeD	17/47 (36)	2.70 (1.02; 7.08)	2.68 (1.00; 7.14)	12/47 (26)	2.29 (0.78; 6.73)	2.11 (0.69; 6.40)			
	classical CeD	8/46 (17)			6/46 (13)					
Age	35+	12/54 (22)	0.57 (0.23; 1.44)	0.67 (0.26; 1.76)	7/54 (13)	0.38 (0.13; 1.09)	0.43 (0.14; 1.28)			
	16–34	13/39 (33)			11/39 (28)					
Gender	M	5/17 (29)	1.17 (0.36; 3.73)	1.06 (0.32; 3.53)	4/17 (24)	1.36 (0.39; 4.81)	1.29 (0.34; 4.86)			
	F	20/76 (26)			14/76 (18)					

Note. Estimates represent the log odds of “score/symptoms worse/not varied = 1” vs. “score/symptoms worse/not varied = 0”. * *n* = number of patients with not improved analysed condition.

## Data Availability

The data presented in this study are available on request from the corresponding author.

## Supplementary Materials

The following are available online at https://www.mdpi.com/article/10.3390/nu13093233/s1, Table S1: Changes in the number of GI and EI symptoms considering follow-ups at T1 and T2; Table S2: Stratified analysis considering the type of CeD and adherence to a GFD (T0-T1); Table S3: Stratified analysis considering sex and age (T0-T1); Table S4: Delta T1-T0 stratified; Table S5: multiple comparisons between T0, T1 and T2 (*n* = 55).
