# Supplementary material for: Quality of Life and Psychological Disorders in Coeliac Disease: A Prospective Multicentre Study
Source: Nutrients. 2021 Sep 16;13(9):3233. doi: 10.3390/nu13093233 (PMC8470791; doi:10.3390/nu13093233)
Supplement: Supplementary file 1 [file nutrients-13-03233-s001.zip › nutrients-1366321-supplementary.pdf]

Supp Table S1. Changes in the number of GI and EI symptoms considering follow-ups at T1 and T2

		T1-T0 (n=93)		T2-T0 (n=55)	T1-T0 (n=55)	T2-T1 (n=55)
		n° patients (%)		n° patients (%)	n° patients (%)	n° patients (%)
**GI symptoms**	**reduction**	**68 (73,1)**	**reduction**	**37 (67,3)**	**42 (76,4)**	**9 (16,4)**
	no difference	17 (18,3)	no difference	13 (23,6)	8 (14,5)	29 (52,7)
	increase	8 (8,6)	increase	5 (9,1)	5 (9,1)	17 (30,9)
**EI symptoms**	**reduction**	**75 (80,7)**	**reduction**	**45 (81,8)**	**44 (80,0)**	**16 (29,1)**
	no difference	14 (15,1)	no difference	9 (16,4)	9 (16,4)	28 (50,9)
	increase	4 (4,2)	increase	1 (1,8)	2 (3,6)	11 (20,0)

Supp Table S2. Stratified analysis considering the type of CeD and adherence to a GFD (T0-T1)

	**Classical (n=46)**						**p-value***
	**T0**			**T1**			
	**min-max**	**median**	**1st-3rd qu.**	**min-max**	**median**	**1st-3rd qu.**	
BDI	0.0-38.0	11.0	5.0-18.0	0.0-29.0	6.0	3.0-15.0	**0.009**
STAY 1	21.0-75.0	42.0	35.0-52.0	21.0-65.0	34.0	29.0-45.5	**0.008**
STAY 2	23.0-74.0	44.0	37.0-50.0	20.0-72.0	38.0	32.5-50.5	**0.005**
HADS-A	0.0-20.0	8.0	5.0-12.8	1.0-19.0	6.0	3.3-10.0	**0.014**
HADS-D	2.0-20.0	6.0	4.0-9.0	1.0-14.0	5.0	3.0-8.0	0.166
SF36	35.5-742.0	386.8	280.5-542.0	152.0-764.0	579.0	390.8-700.2	**0.002**
	**Non-classical (n=47)**						**p-value***
	**T0**			**T1**			
	**min-max**	**median**	**1st-3rd qu.**	**min-max**	**median**	**1st-3rd qu.**	
BDI	0.0-33.0	8.0	4.0-14.5	0.0-38.0	7.0	2.0-9.8	**0.023**
STAY 1	23.0-70.0	38.0	31.0-48.0	22.0-73.0	36.0	29.0-44.3	0.608
STAY 2	27.0-73.0	42.0	33.0-52.0	24.0-71.0	39.0	32.3-47.8	0.206
HADS-A	2.0-16.0	6.0	4.0-9.0	0.0-18.0	6.0	3.3-9.0	0.353
HADS-D	1.0-16.0	5.0	3.5-7.0	1.0-15.0	4.0	3.0-6.0	0.068
SF36	117.0-764.0	523.8	351.4-628.5	133.0-772.0	593.4	510.7-658.4	**0.008**
	**Adherent to GFD (n=78)**						**p-value***
	**T0**			**T1**			
	**min-max**	**median**	**1st-3rd qu.**	**min-max**	**median**	**1st-3rd qu.**	
BDI	0.0-38.0	10.0	5.0-16.0	0.0-38.0	6.0	2.0-13.0	**0.000**
STAY 1	21.0-75.0	38.0	33.0-50.0	21.0-73.0	35.0	29.0-41.8	**0.005**
STAY 2	23.0-73.0	43.0	35.3-51.8	20.0-71.0	38.0	32.0-48.0	**0.001**
HADS-A	0.0-20.0	7.0	4.3-11.0	0.0-18.0	6.0	3.0-9.0	**0.003**
HADS-D	1.0-20.0	5.0	4.0-8.0	1.0-15.0	4.0	3.0-7.0	**0.007**
SF36	35.5-764.0	423.6	319.9-573.2	133.0-772.0	594.3	467.1-689.5	**0.000**
	**Non adherent to GFD (n=12)**						**p-value***
	**T0**			**T1**			
	**min-max**	**median**	**1st-3rd qu.**	**min-max**	**median**	**1st-3rd qu.**	
BDI	0.0-25.0	8.0	4.5-17.0	0-29.0	9.5	6.8-13.5	0.759
STAY 1	24.0-62.0	42.5	38.3-46.5	29-65.0	44.0	32.0-53.0	0.823
STAY 2	31.0-74.0	43.0	36.5-46.5	29-72.0	39.5	36.5-53.5	0.688
HADS-A	2.0-17.0	9.0	5.8-10.3	0-19.0	9.5	5.5-11.3	0.438
HADS-D	3.0-11.0	6.0	4.0-6.3	2-13.0	6.0	5.0-7.0	0.384
SF36	177.3-681.0	633.0	495.4-649.8	244.3-771.0	579.2	492.1-685.0	0.831

* Wilcoxon test

Supp Table S3. Stratified analysis considering sex and age (T0-T1)

	Males (n=17)						p-value*
	T0			T1			
	min-max	median	1st-3rd qu.	min-max	median	1st-3rd qu.	
BDI	0.0-28.0	8.0	4.0-11.0	0.0-38.0	6.0	0.0-13.0	0.675
STAY 1	24.0-63.0	38.0	34.0-43.0	23.0-73.0	30.0	29.0-40.5	0.232
STAY 2	24.0-66.0	41.0	35.0-44.0	20.0-71.0	34.0	32.0-49.0	0.393
HADS-A	0.0-13.0	6.0	3.0-8.0	0.0-18.0	4.0	1.0-8.0	0.465
HADS-D	2.0-16.0	5.0	3.0-8.0	1.0-14.0	5.0	3.0-6.0	0.584
SF36	161.5-764.0	497.7	326.0-656.7	133.0-749.0	659.0	464.2-710.0	0.207
	**Females (n=76)**						**p-value***
	**T0**			**T1**			
	**min-max**	**median**	**1st-3rd qu.**	**min-max**	**median**	**1st-3rd qu.**	
BDI	0.0-38.0	10.0	5.0-17.8	0.0-37.0	6.0	3.0-13.5	**0.000**
STAY 1	21.0-75.0	39.0	32.5-50.0	21.0-65.0	36.0	29.0-45.5	**0.047**
STAY 2	23.0-74.0	44.0	34.5-52.0	22.0-72.0	39.0	33.0-48.3	**0.006**
HADS-A	0.0-20.0	7.0	5.0-11.3	0.0-19.0	6.0	4.0-10.0	**0.026**
HADS-D	1.0-20.0	6.0	4.0-8.0	2.0-15.0	4.0	3.0-7.0	**0.023**
SF36	35.5-742.0	467.0	330.2-578.8	187.0-772.0	580.2	472.1-668.0	**0.000**
	**16-34 (n=39)**						**p-value***
	**T0**			**T1**			
	**min-max**	**median**	**1st-3rd qu.**	**min-max**	**median**	**1st-3rd qu.**	
BDI	0.0-33.0	11.0	5.0-16.5	0.0-37.0	7.0	1.0-12.5	**0.004**
STAY 1	24.0-75.0	40.5	30.0-52.0	22.0-65.0	36.0	29.0-45.0	0.088
STAY 2	27.0-73.0	43.0	40.0-52.0	24.0-63.0	41.0	32.5-49.0	0.137
HADS-A	2.0-18.0	8.0	4.0-12.0	0.0-19.0	6.0	2.5-10.0	**0.025**
HADS-D	2.0-14.0	5.0	3.0-8.0	1.0-15.0	4.0	3.0-7.0	0.109
SF36	142.5-764.0	484.3	341.2-590.2	202.5-772.0	582.8	470.7-696.1	**0.023**
	**35+ (n=54)**						**p-value***
	**T0**			**T1**			
	**min-max**	**median**	**1st-3rd qu.**	**min-max**	**median**	**1st-3rd qu.**	
BDI	0.0-38.0	8.0	4.0-14.8	0.0-38.0	6.0	3.0-14.0	**0.033**
STAY 1	21.0-72.0	38.5	34.3-45.5	21.0-73.0	36.0	30.0-44.0	0.116
STAY 2	23.0-74.0	43.0	35.0-50.0	20.0-72.0	38.0	32.3-48.5	**0.014**
HADS-A	0.0-20.0	7.0	5.0-10.0	1.0-18.0	5.0	4.0-10.0	0.259
HADS-D	1.0-20.0	6.0	4.0-7.8	1.0-14.0	5.0	3.0-7.0	0.118
SF36	35.5-742.0	467.0	326.0-576.5	133.0-764.0	611.5	453.1-679.0	**0.001**

* Wilcoxon test

Supp Table S4. Delta T1-T0 stratified

	Males (N=17)			Females (N=76)			p-value*
	1st qu.	median	3rd qu.	1st qu.	median	3rd qu.	
BDI	-4.0	0.0	2.0	-7.0	-3.0	1.0	0.311
STAY 1	-10.0	-3.0	4.0	-9.8	-2.0	3.0	0.836
STAY 2	-10.0	-3.0	5.0	-10.0	-2.0	1.0	0.668
HADS-A	-2.0	-2.0	2.0	-3.5	-1.0	1.5	0.908
HADS-D	-4.0	-1.0	3.0	-3.0	-1.0	1.0	0.724
SF36	-65.0	57.0	155.0	-18.4	53.2	174.8	0.727
	**16-34 (n=39)**			**35+ (n=54)**			**p-value***
	1st qu.	median	3rd qu.	1st qu.	median	3rd qu.	
BDI	-7.5	-3.0	1.5	-5.0	-3.0	1.0	0.358
STAY 1	-14.5	-3.0	3.8	-9.0	-2.0	3.0	0.364
STAY 2	-10.0	-2.0	2.0	-10.0	-2.0	1.0	0.614
HADS-A	-4.5	-2.0	1.5	-3.0	0.0	2.0	0.272
HADS-D	-3.5	-1.0	1.0	-3.0	-0.5	1.3	0.990
SF36	-31.8	27.3	153.9	-3.6	71.8	178.0	0.596
	**Classical (n=46)**			**Non classical (n=47)**			**p-value***
	1st qu.	median	3rd qu.	1st qu.	median	3rd qu.	
BDI	-7.0	-3.0	1.0	-5.0	-3.0	1.8	0.613
STAY 1	-11.0	-4.0	1.0	-9.3	-0.5	9.0	0.158
STAY 2	-10.0	-3.0	0.8	-8.5	-2.0	3.0	0.260
HADS-A	-5.8	-2.0	2.0	-2.0	0.0	1.0	0.113
HADS-D	-3.0	0.0	2.0	-3.8	-1.0	1.0	0.930
SF36	-15.9	125.9	223.0	-27.8	35.9	133.5	0.265
	**Adherent to GFD (N=78)**			**Not adherent to GFD (N=12)**			**p-value***
	1st qu.	median	3rd qu.	1st qu.	median	3rd qu.	
BDI	-7.0	-3.5	1.0	-2.5	1.0	3.5	0.058
STAY 1	-10.0	-3.0	3.0	-6.5	1.0	7.5	0.189
STAY 2	-10.0	-3.0	1.0	-2.0	1.0	3.5	0.099
HADS-A	-4.0	-1.0	1.0	-0.5	1.0	2.0	**0.050**
HADS-D	-3.8	-1.0	1.0	-0.3	1.0	3.0	0.062
SF36	-14.3	57.0	202.3	-99.8	-68.8	93.9	**0.029**

* Mann-Whitney test

Supp Table S5. multiple comparisons between T0, T1 and T2 (n=55)

	**T0**		**T1**		**T2**		**T0-T1**	**T1-T2**	**T0-T2**
	**Median**	**1st-3rd Qu.**	**Median**	**1st-3rd Qu.**	**Median**	**1st-3rd Qu.**	p-value*	P-value*	P-value*
BDI	8,0	5,0-16,5	6,0	2,0-11	4,0	1,0-8,0	**0.003**	0.060	**0.000**
STAY 1	38,0	31,0-50,5	34,0	28,0-41,8	32,0	28,0-39,0	0.144	**0.015**	**0.000**
STAY 2	43,0	34,0-51,5	38,0	31,8-45,8	36,5	30,3-44,0	0.063	**0.006**	**0.000**
HADS-A	7,0	4,0-11,0	4,5	3,0-8,8	4,0	3,0-7,0	**0.030**	0.204	**0.000**
HADS-D	5,0	4,0-8,0	4,0	3,0-7,0	4,0	3,0-6,8	**0.045**	1.000	**0.003**
SF36	417,8	294,8-560,5	608,2	472,8-696,5	612,8	480,7-704,2	**0.000**	0.141	**0.000**

* Wilcoxon test; p-values adjusted (Dunn-Bonferroni) for multiple comparison
